# The main forest species encountered in southern and central Benin, West Africa

**DOI:** 10.3897/BDJ.12.e129134

**Published:** 2024-09-05

**Authors:** Gorgias Aikpon, Kourouma Koura, Jean C. Ganglo

**Affiliations:** 1 Laboratory of Forest Sciences, Abomey-Calavi, Benin Laboratory of Forest Sciences Abomey-Calavi Benin

**Keywords:** dataset, biodiversity, forest species, southern Benin, west Africa

## Abstract

**Background:**

The south of Benin, a country in West Africa, is still home to remnants of dense forests that benefit from a particularly rainy sub-equatorial climate, with annual rainfall of up to 1,200 mm. These forest ecosystems are an integral part of the West African forest block, which stretches from Liberia to Togo. However, despite their richness and ecological importance, these forests are unfortunately subject to strong human pressures, particularly from slash-and-burn agriculture, intensive logging and the growing urbanisation of coastal areas. Preserving these forests is crucial, however, as they are home to remarkable plant and animal biodiversity, with many endemic species. What's more, these forests play an essential role in regulating the local climate, protecting soil and water resources, as well as providing local populations with a vital source of energy wood, non-timber forest products and support for their traditional agricultural practices. Faced with these conservation challenges, identifying and characterising the main tree species found in the forests of southern and central Benin, forest species and their ecology is an essential prerequisite for implementing sustainable management and restoration strategies for these threatened forest ecosystems in southern Benin. This work aims to identify and draw attention to the different forest species, specially tree forest species present in southern and central Benin.

**New information:**

The dataset provides information on forest species found in southern and central Benin, West Africa. This dataset is extremely useful for forestry research, as it focuses mainly on the various forest species of major importance. It can be used as a basis for characterising individuals or populations of species, based on their abundance in relation to anthropogenic pressure and changes in environmental conditions.

These species are characteristic of forests and, above all, are of particular interest both to populations and to managers of protected areas. Field collections were initiated in 2007 as part of natural forest inventory work. The data collected have been completed by various field works that followed this work on forest species in southern Benin. The latest version of the dataset is publicly and freely accessible on the GBIF website at the address https://www.gbif.org/dataset/aff3a10a-a86b-4eff-98e4-d63f92fd6f7e.

It should be noted that the fact that the collection and monitoring were carried out in southern Benin, a region known for its great diversity of species, over a period of 10 years making these data particularly relevant information to study the effects of climate change and human pressure on ecosystems in this area.

## Introduction

The history of flora research in Benin spans several centuries and has gone through different phases, reflecting the evolution of scientific approaches and political changes in the country (Amoussou et al. 2016). The first botanical explorations in Benin (then known as Dahomey) date back to the colonial period (Arouna et al. 2016). European explorers and naturalists, mainly French, began documenting the region's flora at the end of the 19^th^ century (Avakoudjo et al. 2014). These early expeditions were often motivated by the search for plants of economic or medicinal interest, as well as by scientific curiosity (Lambin et al. 2003).

In the early 20^th^ century, efforts to collect and document plants intensified (Mama et al. 2013). Botanists such as Auguste Chevalier led major expeditions, making a significant contribution to our knowledge of West African flora, including that of Benin (Romeiras et al. 2018). This work laid the foundations for the first regional floras. After Benin gained independence in 1960, botanical research was gradually taken over by Beninese scientists (Wood et al. 2004). The University of Abomey-Calavi, founded in 1970, became an important centre for national botanical research (Mama et al. 2013a). In the 1990s and 2000s, research into Benin's flora diversified and deepened (Chabi-Boni et al. 2019). More specialised studies were carried out on specific taxonomic groups, plant ecology and ethnobotany (Amoussou et al. 2016). More recently, botanical research in Benin has focused on contemporary issues such as the conservation of biodiversity, the impact of climate change on flora and the development of local plant resources (Zakari et al. 2018). Major efforts have been devoted to creating and updating national herbaria, which are essential tools for research and conservation (Zakari et al. 2015). Today, flora research in Benin continues to evolve, with an increasing emphasis on interdisciplinary approaches (Djossa et al. 2008).

The Republic of Benin is located in the intertropical zone, between parallels 6°30' and 12°30' north latitude and meridians 1° and 3°40' east longitude (Neuenschwander and Toko 2011). With the exception of the north-western part of the Atacora chains and the centre of the hill department, the country is relatively flat. Southern Benin is located in the driest corridor generally referred to as the "Dahomey Gap", which separates the West African rainforest belt into two blocks: the Guinean (or western) and Congolese (eastern) forest blocks (FAO 2005). This Dahomean climatic discontinuity is characterised by pronounced water deficits, resulting in the absence of dense evergreen forest with all its flora and fauna (FAO 2010). The only remaining physiognomic unit is "dense semi-deciduous tropical forest", but this is now fragmented and crumbling (Imorou et al. 2019). The consequences of fragmentation are compounded by the erosion of biological resources caused by human activities such as shifting cultivation and logging (Aoudji and Ganglo 2006). The conservation of plant resources should be an overriding objective in Benin; given the fragmentation of natural environments, it seems that the conservation of specimens from each type of ecosystem is the best approach for safeguarding the completeness of the flora (Akoègninou et al. 2006, De-Souza 2009). In the context of biodiversity conservation, communities rich in rare and threatened species of conservation interest are generally referred to as biodiversity hotspots (IUCN 2000). Particular attention is, therefore, increasingly being paid to the species richness and endemism of sites (Natta et al. 2002). The issue of plant biodiversity conservation in Benin deserves to be revisited in these terms.

It should be noted that the unavailability or lack of data on the distribution of species of particular conservation interest (rare, endemic or threatened) is a real problem. It is interesting to examine whether, in this respect, the number and geographical distribution of existing national parks and reserves allow effective conservation of Benin's flora.

## General description

### Purpose

This database focuses mainly on forest species in southern Benin. All occurrences are entered by several headings: Occurrence ID, CollectionCode, CatalogNumber. The database also includes the name of the institution that coordinated the work, the complete taxonomy, the names of the collectors and many other important information: dates (day, month, year), regions, countries, exact sites, longitudes and latitudes converted to decimal degrees. This base can be used for various studies. Indeed, it can be used in studies that focus on the diversity of forest species (species richness, distribution models), spatial distribution and ecological niche modelling.

### Additional information

Licence CC BY 4.0

## Project description

### Title

Inventory of forest ecosystems in southern and central Benin

### Study area description

**Figure 1.**
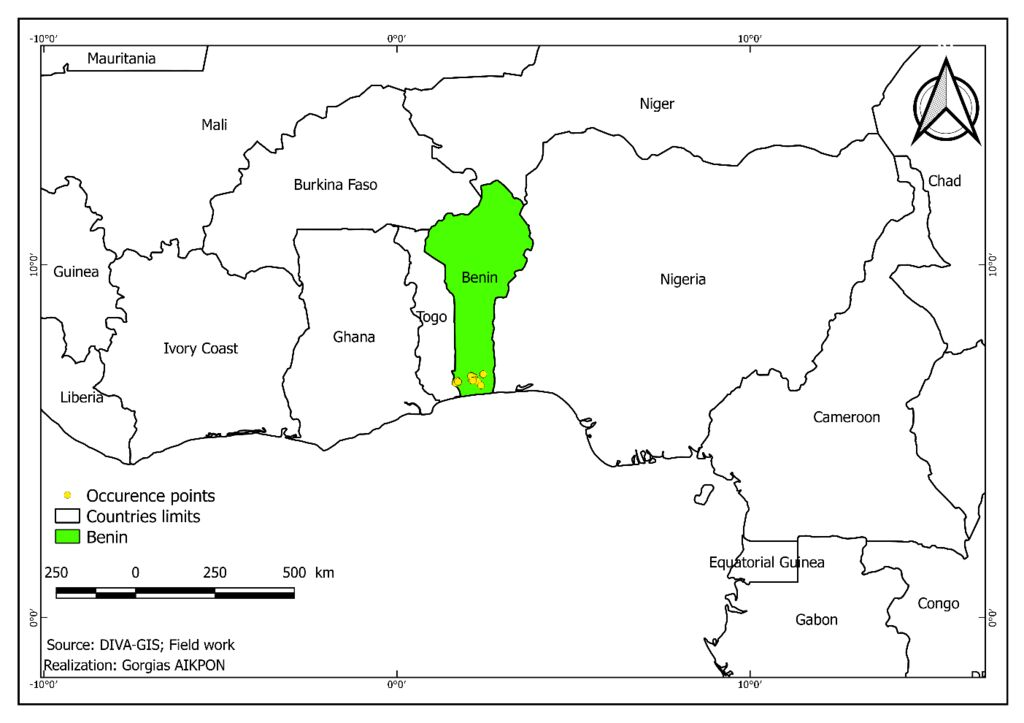
Spatial distribution of the occurrence.

**Figure 2.**
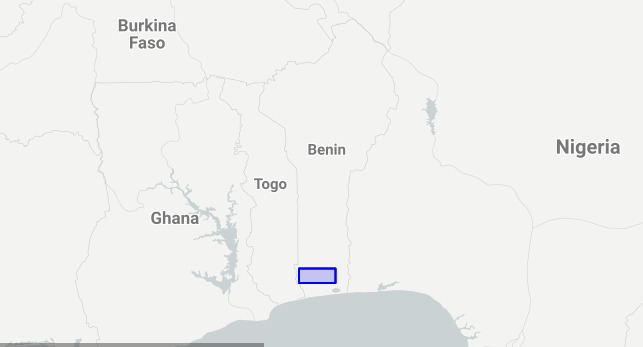
Geographic scope of the dataset.

The 7,126 occurrences of this dataset were collected in South Benin, West Africa (Figs 1, 2)

## Sampling methods

### Sampling description

This database is devoted to forest species found in southern Benin. Forest species ensure the balance of the ecosystems that belong to them and are of great importance for the survival of humanity (Ganglo 2005).

The Republic of Benin covers the major part of the 'Dahomey Gap' which is an interruption of the dense forest on the coast of West Africa. Few biogeographical regions have been the subject of an enigma comparable to the phenomenon of the "Dahomey Gap" in this part of the West African coast where the savannah comes directly into contact with the coastal strips. The climate is particularly dry (less than 1000 mm per year towards the southwest). The vegetation is in the form of a mosaic of forests, savannahs and fields.

Benin has fairly limited forest resources (Awokou et al. 2009). These meagre resources are in the grip of alarming degradation (Aoudji and Ganglo 2006). Indeed, according to the latest figures, Benin is losing an average of 65,000 ha of forest per year (FAO 2005). It is, therefore, important to better control the available forest resources.

These are observational data collected during fieldwork. The forest inventories were carried out by setting up 1 hectare plots. This rigorous method provided data on the structure and composition of the forests. For this approach, careful planning was carried out, where the objectives of identifying forest tree species were clearly defined. The occurrence data bring together a range of forest inventory work carried out. The number and distribution of plots were determined according to the variability of the terrain and the resources available.

In the field, the plots were set up by precisely locating the starting points using a GPS. A 100-metre square was then marked out, representing an exact area of one hectare. The corners are visibly marked, usually with coloured stakes or ribbons. To facilitate the inventory work, the plot can be subdivided into sub-plots.The measurement and survey phase is one of the main step of the inventory. All trees with a diameter at breast height (DBH) equal to or greater than 10 centimetres are identified, measured and recorded. The DBH is measured at 1.30 metres from the ground using a tape measure or a forestry compass. The total height of the tree and the height of the bole are estimated, often using a clinometer or dendrometer.

Data were collected systematically, either on standardised field sheets or using tablets equipped with specialised software. Each tree was given a unique number and all the information about it are carefully recorded. As well as trees, other elements of the forest ecosystem were studied. Regeneration was assessed on sub-plots and botanical samples were taken for later identification if necessary. Processing the data collected was the final stage of the inventory. The information was entered into a database, cleaned and checked. Calculations were made to obtain dendrometric parameters.

**Figure 3.**
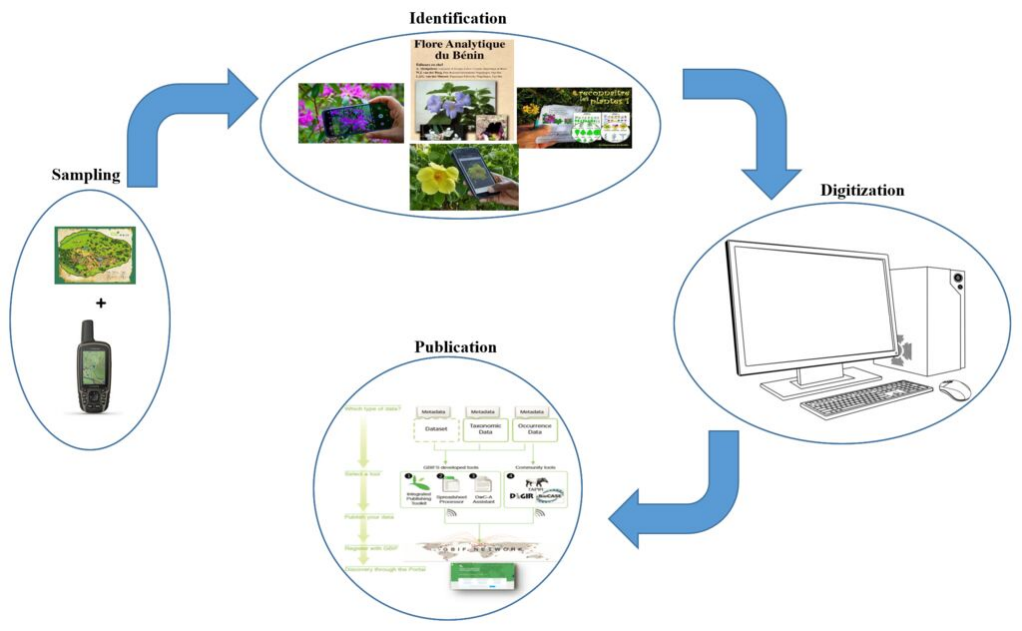
Synoptic of the procedure used to generate the dataset.

Although time-consuming and resource-intensive, this methodology provides a good characterisation of forest ecosystems. It provides essential data for sustainable forest management, biodiversity conservation and the study of long-term forest dynamics. The geographic coordinates were taken with a GPS. It should be noted that, once the data were collected in the field, all the information was carefully digitised. A correct data entry verification followed. Scientific names have also been checked for accuracy (Fig. 3).

## Geographic coverage

### Description

This database is devoted to forest species found in southern Benin (Fig. 2).

### Coordinates

6.901561 and 6.579434 Latitude; 1.650389 and 2.44661 Longitude.

## Taxonomic coverage

### Description

**Figure 4.**
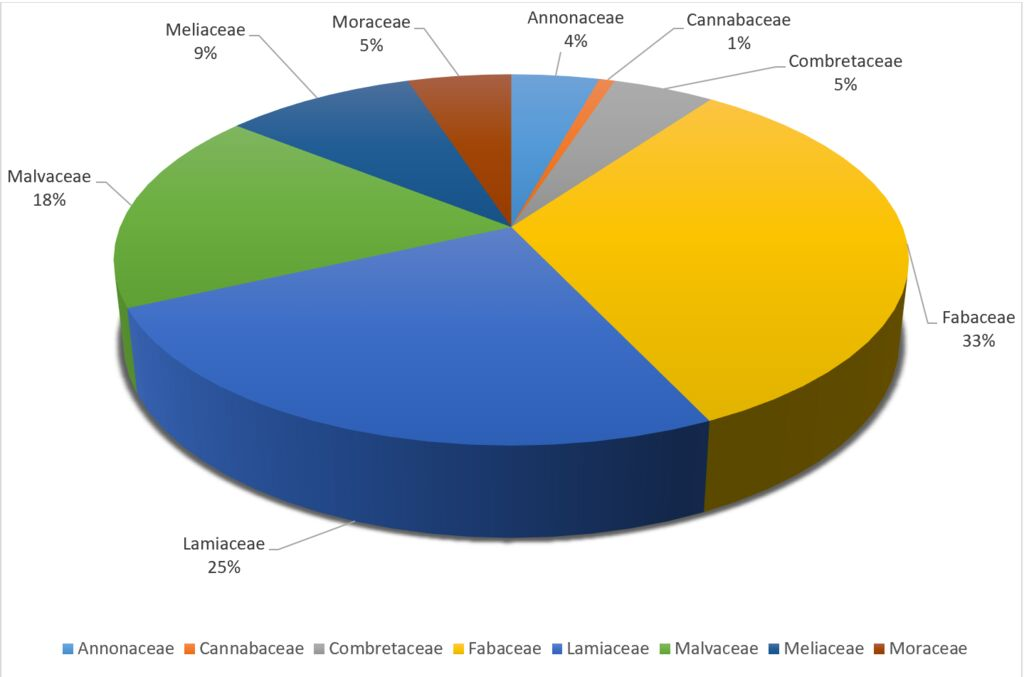
Taxonomic coverage of tree species at the family level in the dataset.

The identifications are reflected in the analytical flora of Benin (Akoègninou et al. 2006). In total, eight (8) families have been designated. These are: Annonaceae, Cannabaceae, Combretaceae, Fabaceae, Lamiaceae, Malvaceae, Meliaceae and Moraceae (Fig. 4). The Annonaceae family, also known as the Annone family, comprises mainly tropical trees and shrubs. It is known for its edible fruits, such as cherimoya and soursop (Adeoye and Ewete 2010). Plants in this family generally have simple, alternate leaves and radially symmetrical flowers with numerous stamens. The bark of some species is used in traditional medicine (Mama et al. 2013b). The Cannabaceae family now includes several genera of trees. Plants in this family often have palmate or toothed leaves and inconspicuous flowers (Aina et al. 2009). The Combretaceae family mainly comprises tropical trees and shrubs. Plants in this family often have simple leaves and winged fruits. Some species are used in traditional medicine and agroforestry (Aina et al. 2009). The Fabaceae family is one of the largest families of flowering plants and it includes trees, shrubs and grasses, many of which are of economic importance (Akinwumi et al. 2007). Plants in this family are characterised by pod-shaped fruits and often compound leaves (Amoussou et al. 2012). The Lamiaceae family, formerly known as Labiatae, includes many aromatic herbs such as mint, thyme and rosemary. Plants in this family often have square stems, opposite leaves and bilabiate flowers (Aku et al. 1998). The Malvaceae family includes plants as diverse as cocoa and baobab (Alles et al. 2010). Plants in this family often have alternate leaves and five-petalled flowers with numerous fused stamens (Adeoye and Ewete 2010). The Meliaceae family comprises mainly tropical trees, many of which are important for their precious wood, such as mahogany (Allison et al. 2013). Plants in this family generally have pinnate compound leaves and capsule-shaped fruits. Some species are used in traditional medicine (Álvarez et al. 2008). The Moraceae family includes trees, shrubs and a few herbs, such as fig and mulberry (Anita et al. 2012). Plants in this family often have a milky latex and complex fruits and some species are important for agroforestry and horticulture (Braga and Valle 2007). These families represent a great diversity of plants, each with unique characteristics and ecological and economic importance.

## Temporal coverage

### Notes

The occurrences of this database covers the period from 2007 to 2017. It is a compilation of forest inventories carried out during this period.

## Usage licence

### Usage licence

Creative Commons Public Domain Waiver (CC-Zero)

### IP rights notes

Licence CC BY 4.0

## Data resources

### Data package title

The main forest species encountered in southern and central Benin, West Africa.

### Resource link

https://doi.org/10.15468/sr46ah

### Alternative identifiers

https://www.gbif.org/dataset/aff3a10a-a86b-4eff-98e4-d63f92fd6f7e

### Number of data sets

1

### Data set 1.

#### Data set name

The main forest species encountered in southern and central Benin, West Africa.

#### Data format

csv

#### Character set

Arial

#### Download URL

https://www.gbif.org/dataset/aff3a10a-a86b-4eff-98e4-d63f92fd6f7e

#### Data format version

csv

#### Description

The dataset gathers information on the major forest species present in the southern region of Benin, a country in West Africa. The dataset therefore includes several species from different families. It includes a total of 27 different species, with 7126 data entries relating to them (Djeho 2021).

**Data set 1.** 

Column label	Column description
occurrenceID	An identifier for the occurrence.
institutionCode	The name or acronym in use by the institution.
collectionID	An identifier for the collection or dataset from which the data were derived.
collectionCode	The name, acronym, coden or initialism identifying the collection or dataset from which the record was derived.
catalogNumber	An identifier for the record within the dataset or collection.
sex	The sex of the biological individual (s) represented in the Occurrence.
kingdom	The full scientific name of the kingdom in which the taxon is classified.
phylum	The full scientific name of the phylm or division in which the taxon is classified.
class	The full scientific name of the class in which the taxon is classified.
order	The full scientific name of the order in which the taxon is classified.
family	The full scientific name of the family in which the taxon is classified.
genus	The full scientific name of the genus in which the taxon is classified.
subgenus	The full scientific name of the subgenus in which the taxon is classified.
specificEpithet	The name of the first or species epithet of scientificName.
infraspecificEpithet	The name of the lowest or terminal infraspecific of the scientificName, excluding any rank designation.
scientificName	The full scientificName name, with authorship and date information if known.
scientificNameAuthorship	The authorship information for the scientificName formatted according the conventions of the applicable nomenclaturalCode.
modified	The most recent date-time on which the resource was changed.
taxonRank	The taxonomix rank of the most specific name in the scientificName.
dateIdentified	The date on which the subject was identified as representing the taxa.
identifiedBy	A list of names of people, groups or organisations who assigned the Taxon to the subject.
typeStatus	A list of nomenclatural types applied to the subject.
continent	The name of the continent in which the Location occurs.
waterBody	The name of the waterbody in which the Location occurs.
country	The name of country or major administrative unit in which the Location occurs.
stateProvince	The name of the next smaller administrative region than country.
locality	The specific description of the place.
decimalLatitude	The geographic latitude in decimal degrees.
decimalLongitude	The geographic longitude in decimal degrees.
coordinatePrecision	A decimal representation of the presicion of the coordinates given in the decimalLatitude and decimalLongitude.
minimumElevationInMetres	The lowest limit of the range of elevation in metres.
maximumElevationInMetres	The upper limit of the range of elevation in metres.
minimumDepthInMeters	The lesser depht of a range of depht below the local surface, in meters.
maximumDepthInMetres	The greater depth of a range of depth below the local surface, in metres.
basisOfRecord	The specific nature of the data record.
eventDate	The date-time or interval during which an Event occurred.
Year	The four-digit year in which the Event occurred, according to the Commen Era Calendar.
Month	The ordinal month in which the Event occurred.
Day	The integer day of the month on which the Event occurred.
habitat	A category or description of the habitat in which the Event occurred.
fieldNumber	An identifier given to the Event in the field.
recordedBy	A list of names of people, groups or organisations responsible for recording the original Occurrence.
samplingProtocol	The name of, reference to, or description of the method or protocol use during an Event.
associatedMedia	A list of identifiers of media associated with the Occurrence.
eventRemarks	Comments or notes about the Event.

## Additional information

### Conflict Of Interests

The authors have declared no conflict of interests.
